# An osteopenic/osteoporotic phenotype delays alveolar bone repair

**DOI:** 10.1016/j.bone.2018.04.019

**Published:** 2018-04-25

**Authors:** Chih-Hao Chen, Liao Wang, U. Serdar Tulu, Masaki Arioka, Melika Maghazeh Moghim, Benjamin Salmon, Chien-Tzung Chen, Waldemar Hoffmann, Jessica Gilgenbach, John B. Brunski, Jill A. Helms

**Affiliations:** a Craniofacial Research Center, Department of Plastic and Reconstructive Surgery, Chang Gung Memorial Hospital, Chang Gung University School of Medicine, Taoyuan 33305, Taiwan; b Division of Plastic and Reconstructive Surgery, Department of Surgery, Stanford University School of Medicine, Stanford, CA 94305, USA; c State Key Laboratory of Oral Diseases, West China Hospital of Stomatology, Sichuan University, Chengdu 610041, China; d Department of Clinical Pharmacology, Faculty of Medical Sciences, Kyushu University, Fukuoka 812-8582, Japan; e University College London Medical School, University College London, London WC1E 6BT, UK; f Paris Descartes University - Sorbonne Paris Cité, EA 2496 - Orofacial Pathologies, Imaging and Biotherapies Lab and Dental Medicine Department, Bretonneau Hospital, HUPNVS, AP-HP, Paris, France; g Department of Plastic and Reconstructive Surgery, Chang Gung Memorial Hospital at Keelung, Keelung 20401, Taiwan; h Nobel Biocare Services AG P.O. Box, CH-8058 Zürich-Flughafen, Switzerland

**Keywords:** Rats, Ovariectomy, Tooth extraction, Osteotomy, Alveolar bone, Computer models

## Abstract

Aging is associated with a function decline in tissue homeostasis and tissue repair. Aging is also associated with an increased incidence in osteopenia and osteoporosis, but whether these low bone mass diseases are a risk factor for delayed bone healing still remains controversial. Addressing this question is of direct clinical relevance for dental patients, since most implants are performed in older patients who are at risk of developing low bone mass conditions. The objective of this study was to assess how an osteopenic/osteoporotic phenotype affected the rate of new alveolar bone formation. Using an ovariectomized (OVX) rat model, the rates of tooth extraction socket and osteotomy healing were compared with age-matched controls. Imaging, along with molecular, cellular, and histologic analyses, demonstrated that OVX produced an overt osteoporotic phenotype in long bones, but only a subtle phenotype in alveolar bone. Nonetheless, the OVX group demonstrated significantly slower alveolar bone healing in both the extraction socket, and in the osteotomy produced in a healed extraction site. Most notably, osteotomy site preparation created a dramatically wider zone of dying and dead osteocytes in the OVX group, which was coupled with more extensive bone remodeling and a delay in the differentiation of osteoblasts. Collectively, these analyses demonstrate that the emergence of an osteoporotic phenotype delays new alveolar bone formation.

## 1. Introduction

Low bone mass diseases including osteopenia/osteoporosis decrease bone quantity, which in turn impacts bone quality [1,2]. An osteoporotic skeleton is characterized by a decrease in bone volume and bone mineral density, making the skeleton more fragile [3] and therefore prone to fracture [4]. Osteoporosis is also associated with an increase in marrow adipogenesis, commensurate with a loss in osteoblastogenesis [5,6].

Whether osteopenia/osteoporosis is a risk factor for delayed bone healing, however, is unclear. For example, rats subjected to an ovariectomy (OVX), which reliably produces an osteoporotic phenotype [7], exhibit slower healing of tooth extraction sockets compared to age-matched non-OVX rats [8,9]. Rodent models of long bone fractures also exhibit slower healing (reviewed in [10]).

Clinical data are less clear. In some meta-analyses, bone healing around dental implants appeared to be unaffected in osteoporotic patients [11,12] But in other analyses, peri-implant bone loss was more severe in osteoporotic patients ([13]; reviewed in [14]). Most studies fail to report whether patients are actively taking anti-osteoporotic medications e.g., bisphosphonates, which may impact bone remodeling rates [15,16]. An additional confounding variable is that most patients are older, and it is inherently challenging to separate the effects of osteoporosis from other age-related diseases that can impact the skeleton. Understanding whether low bone mass diseases impact alveolar bone osteogenesis has direct clinical relevance for dental patients, since most implants are performed in individuals over the age of 50 [17,18] and this group is at risk for developing osteopenia and osteoporosis [19].

These data served as a starting point for our study: using the same OVX rat model we undertook a series of experiments to uncover the mechanisms whereby an osteoporotic phenotype impacts bone healing. We used both extraction socket repair and osteotomy repair as injury models, and employed a battery of molecular, cellular, and imaging tools to reveal how osteoporosis negatively impacts alveolar bone healing.

## 2. Materials & methods

### 2.1. Animals, experimental plan, and surgeries

Stanford APLAC approved all procedures, which conform to ARRIVE guidelines. In total, forty-eight 6-week-old (young) and five 12-month-old (aged) female rats (Wistar, Charles River) were used in this study. Three surgeries were carried out: an ovariectomy (OVX); a tooth extraction; and an osteotomy. Each surgery is described below. In all cases, anesthesia was reached via intraperitoneal injection of Ketamine (100 mg/kg) and Xylazine (10 mg/kg); and analgesia via subcutaneous injection of Buprenorphine-SR (0.5 mg/kg). Rats were randomly assigned to experimental groups that are outlined in Table 1.

OVX surgery followed previous guidelines where a dorsal midline incision was made between the mid-back and tail base. The peritoneal cavity was accessed through bilateral muscle layer incisions, the ovary was identified, the connection between the fallopian tube and the uterine horn was suture-ligated, and the ovary was removed. Wounds were closed layer by layer.

Bilateral maxillary first molars (e.g., M1) extractions were performed using micro-forceps. After extraction, bleeding was controlled by local compression. Rats recovered in a controlled, heated environment, then were fed a soft food diet for the duration of the experiment and housed in groups of two. Weight changes were < 10%. No adverse events (e.g., uncontrolled pain, infection, prolonged inflammation) were encountered. In Group 1, M1 extraction was performed concurrent with the OVX procedure. In Group 2, M1 extraction was delayed 8 weeks following the OVX. Animals were sacrificed at post-extraction days (PEDs) 0, 1, 7 and 21 for analysis.

Osteotomy site preparation followed previously established procedures [20]. Briefly, osteotomies were produced in the healed M1 extraction site using a low-speed handpiece (KaVo Dental UK; 5333 rpm) using 0.3 mm drill bits (Drill Bit City, NY) with saline irrigation. No adverse events were encountered during the healing process. Tissues were collected at post-osteotomy days (PODs) 0.5 and 7.

### 2.2. Tissue preparation and histology

After harvesting, samples were prepared for histology as described [21]. Briefly, samples were fixed in 4% paraformaldehyde (PFA), decalcified in ethylene diamine tetra-acetic acid (EDTA), dehydrated using an ascending graded ethanol series, and embedded into paraffin blocks for sectioning. Before staining or other histological/cellular activity analysis, all sections were de-paraffinized in Citrisolv (#1601, Decon Labs Inc. PA), and hydrated via a descending graded ethanol series. After staining, sections were dehydrated in a graded series of ethanol and Citrisolv, and subsequently cover-slipped with Permount (#SP15, Fisher Scientific) mounting media.

For Aniline blue staining, slides were treated with a saturated solution of picric acid, followed by a 5% Phosphotungstic acid solution and staining in 1% Aniline blue. Pentachrome staining was performed as described [22]. In brief, after dehydration, slides were stained with 1% Alcian Blue (#A5268, Sigma), Verhoeffs Hematoxylin (#S71299, Fisher Scientific), Sodium Thiosulfate (#14518, Alfa Aesar, MA), Crocein-Scarlet-Acid Fuchsin solution (#22914, Chem Impex International, IL; #F8129, Sigma), 5% Phosphotungstic Acid (#P4006, Sigma) and Saffron (#3801, Harlecon), with washing steps between each stain using ethanol, acetic acid and distilled water. For Picrosirius Red staining, slides were stained with picrosirius solution (0.5 g Sirius red (#35780, Pfaltz & Bauer, Inc., CT) dissolved in 500 mL saturated picric acid solution), and then viewed under polarized light.

### 2.3. Alkaline phosphatase (ALP) and Tartrate-resistant acid phosphatase (TRAP) activities

To detect ALP activity, tissue sections were treated with ALP-detection solution containing BCIP (5-bromo-4-chloro-3-indolyl phosphate; Roche, #11383221001) and NBT (nitro blue tetrazolium chloride; Roche, #11383213001) at 37 °C for 30 min according to the manufacturer’s instructions. TRAP activity was observed using a Leukocyte Acid Phosphatase Staining Kit (catalog #386A-1KT, Sigma-Aldrich, St. Louis, MO). Tissue sections were processed according to the manufacturer’s instructions

### 2.4. Detection of viable/dead cell nuclei

The number and distribution of viable cells were determined using a nuclear stain, DAPI. Slides were mounted with ProLong Gold Antifade Mountant containing DAPI (#P36935, Life Sciences) then viewed under fluorescent light. TUNEL (#11684795910, Roche, Indianapolis, IN) was performed as described by the manufacturer.

To quantify the extent of apoptotic osteocytes due to drilling, TUNEL stained tissue sections of drill sites were analyzed from 4 to 6 different samples taken from trabecular bone. Each section was photographed using a Leica digital image system. The number of TUNEL-labelled osteocytes, corresponding to apoptotic cells, was determined and grouped according to their distance from the cut edge. The corresponding area for each group was then calculated. The number of apoptotic cells per unit area was found by dividing number of apoptotic cells to the corresponding area (cell/mm^2^).

### 2.5. Immunostaining

Immunostaining was performed using standard procedures [23]. In brief, endogenous peroxidase activity was quenched by 3% hydrogen peroxide for 5 min, and then washed in PBS. Slides were blocked with 5% goat serum (S-1000, Vector Labs, CA) for 1 h at room temperature. The appropriate primary antibody was added and incubated overnight at 4 °C, then washed in PBS. The primary antibodies used in this paper are: Osterix (1:1200; ab22552, Abcam), CathepsinK (1:200; ab19027, Abcam), Periostin (1:200; ab14041, Abcam). Samples were incubated with appropriate biotinylated secondary antibodies (Vector BA-x) for 30 min, then washed in PBS. An avidin/biotinylated enzyme complex (Kit ABC Peroxidase Standard Vectastain PK-4000) was added and incubated for 30 min and a DAB substrate Kit (Kit Vector Peroxidase substrate DAB SK-4100) was used to develop the color reaction.

### 2.6. Micro-computed tomography (μCT)

Scanning and analyses followed published guidelines [24]. Three-dimensional μCT imaging was performed at various times after surgery. In brief, samples were fixed in 4% PFA at 4 °C overnight and washed in PBS. During scanning, the samples were kept in 70% ethanol solution. μCT scanning was performed on Viva CT data-acquisition system (Scanco, Brüttisellen, Switzerland) at 10.5 μm voxel size (70 kV, 115 μA and 300 ms integration time). Bone morphometry was performed using the acquisition system’s analysis software (Scanco). Multiplanar reconstruction and volume rendering were carried out using Avizo (FEI, Hillsboro, OR) and ImageJ (NIH, Bethesda, MD) software. Images were organized using Adobe Photoshop and Illustrator (Adobe, San Jose, CA). Results were presented in the form of mean ± standard deviation, with *N* equal to the number of samples analyzed.

### 2.7. Histomorphometric analyses

Histomorphometric measurements were performed using ImageJ software (NIH, Bethesda, MD). To quantify the amount of new bone formation in tooth extraction sockets, Aniline blue-stained histologic sections that spanned the distance from the furcation to the apex were used. Each section was photographed using a Leica digital image system at 20× magnification. To find the percentage of new bone, the number of Aniline Blue^+ve^ pixels within an osteotomy were calculated and divided by the number of the total pixels in the same osteotomy area.

### 2.8. Statistical analyses

Results are presented in the form of mean ± standard deviation. All statistical analyses were performed using the GraphPad website. Histomorphometric results were based on t-tests and unpaired t-tests. Significance was attained at *p* < 0.05 (*), at *p* < 0.01 (**), at *p* < 0.001 (***).

## 3. Results

### 3.1. Ovariectomy produces an osteoporotic phenotype in both appendicular and alveolar bones

**Fig. 1.**
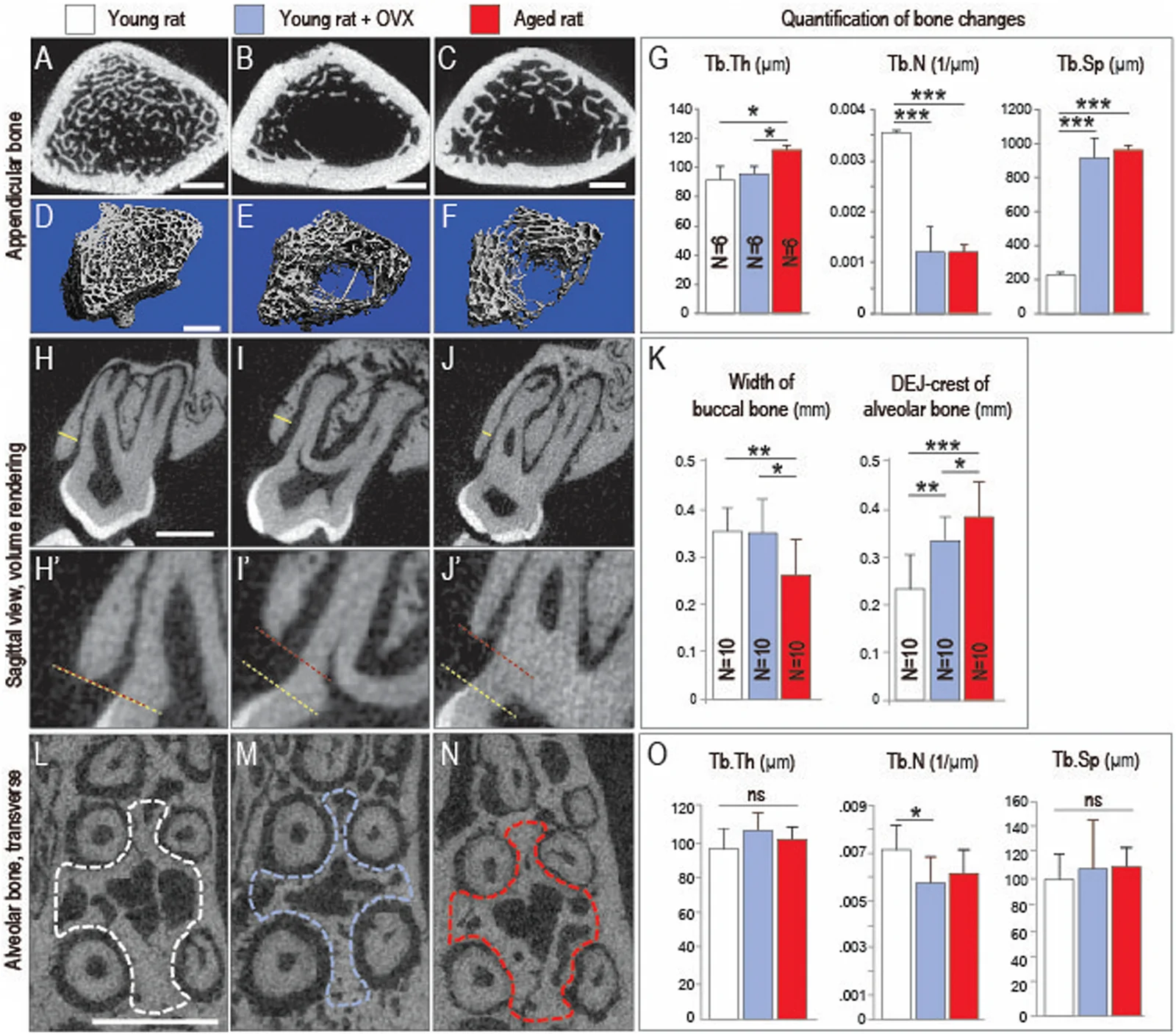
OVX produces both appendicular and alveolar bone loss resembling the bone loss seen in aged animals. Representative transverse μCT sections of distal femur in animals of (A) young, (B) young +OVX, and (C) aged groups. Three-dimensional visualization of trabecular bone in (D) young, (E) young + OVX and (F) aged rats. (G) Quantification of trabecular thickness (Tb.Th), trabecular number (Tb.N) and trabecular separation (Tb.Sp) in the three groups. Representative coronal μCT sections, with yellow lines demonstrating the width of the buccal bone from young (H), young +OVX (I) and aged (J) rats. Higher magnification, with dotted lines demonstrating the height of the alveolar bone crest (red) relative to the dento-enamel junction (DEJ, yellow) in (H′) young, (I′) young + OVX and (J′) aged animals. (K) Quantification of width of the buccal bone and height of the alveolar bone crest relative to DEJ across the different groups. Representative transverse μCT sections of alveolar bone surrounding mxM2, from (L) young, (M) young + OVX, and (N) aged rats; equivalent regions of interest are indicated by dotted lines. (O) Quantification of Tb.Th, Tb.N and Tb.Sp in alveolar bone around second maxillary molar. Abbreviations: Tb.Th, trabecular thickness; Tb.N, trabecular number; Tb.Sp, trabecular separation; DEJ, dento-enamel junction. Scale bars = 1 mm.

Our first objective was to determine if an OVX surgery created an osteopenic/osteoporotic phenotype that was detectable in the jawbones. A cohort of young rats served as a positive control for optimal bone health (Fig. 1A). The same aged young rats that had undergone OVX 8 weeks prior constituted the test group (Fig. 1B), and a cohort of aged (~12-month-old) rats served as a negative control for age-related changes in bone health (Fig. 1C).

Rats were evaluated when they were 15 weeks old. Volume rendering of the distal femur revealed a dense pattern of trabecular bone in the young group (Fig. 1D). In the young + OVX group, trabecular bone volume was significantly reduced (Fig. 1E), comparable to the aged group (Fig. 1F; quantified in G). Trabecular number and trabecular separation in the young + OVX group were also equivalent to that seen in the aged group (Fig. 1G).

Clinical data indicate that with age the buccal plate of maxillary bone thins, and alveolar bone height recedes [25,26] therefore we evaluated these characteristics in the experimental groups. The young rat cohort exhibited the thickest buccal plate (Fig. 1H) and exhibited the most coronal alveolar crest height (Fig. 1H′; quantified in K). In the young + OVX group the thickness of the buccal bone did not differ significantly from the young group (Fig. 1I) but the height of the alveolar crest was significantly receded (Fig. 1I′; quantified in K). In the aged group, the buccal bone was significantly thinner (Fig. 1J), and the alveolar bone showed the maximum resorption (Fig. 1J′; quantified in K). These additional analyses indicated that OVX produced an osteopenic/osteoporotic phenotype affecting both appendicular and craniofacial skeletons, although in the latter the bony changes were more subtle. While the appendicular skeletons of aged and young + OVX rats showed clear evidence of osteopenia/osteoporosis, transverse μCT sections through the maxillary alveolar bone did not (Fig. 1L–N). Quantification of a region of interest in the alveolar bones of the three groups (dotted lines in Fig. 1L–N) showed no significant differences in trabecular thickness and separation (Fig. 1O). Only trabecular number was significantly diminished in the young + OVX group compared to the young group (Fig. 1O).

### 3.2. Extraction socket healing is negatively impacted by emergence of an osteoporotic phenotype

**Fig. 2.**
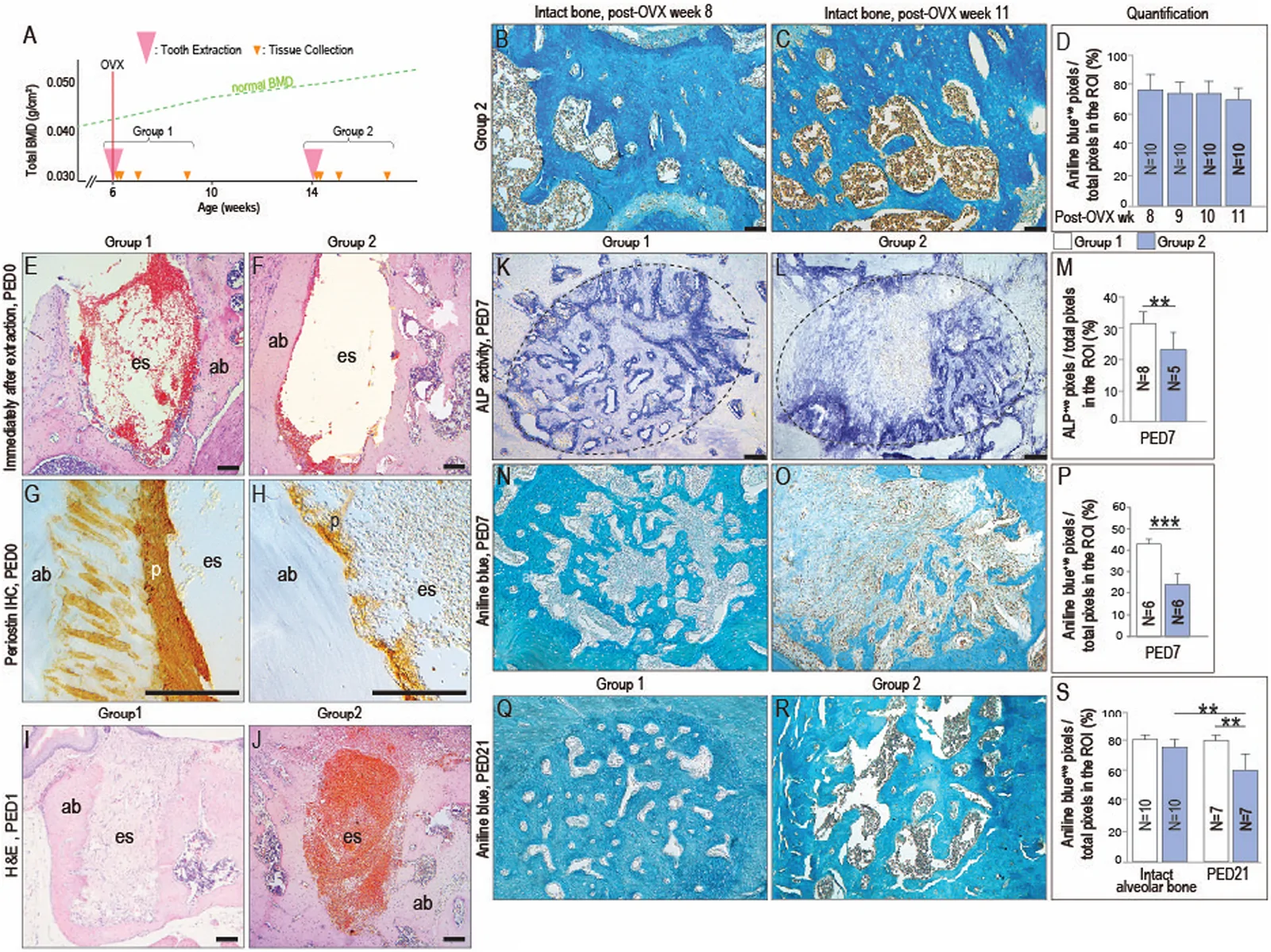
The rate of extraction socket healing depends on the emergence of the OVX phenotype. (A) Schematic of the experimental model where 6-week-old rats underwent OVX and bilateral first molar (M1) extractions. In Group 1, tooth extraction occurred immediately after OVX, followed by sacrifice at post-extraction days (PEDs) 0, 1, 7 and 21. In Group 2 OVX was followed by an 8-week recovery period during which the OVX phenotype developed; afterwards tooth extraction was performed, followed by sacrifice at the same time points. Representative transverse tissue section stained by Aniline blue from intact alveolar bone in Group 2 at (B) post-OVX week 8, and (C) post-OVX week 11. (D) Quantification of Aniline Blue^+ve^ pixels/total pixels in the intact bone from Group 2. Representative sagittal H&E-stained tissue sections from rats in (E) Group 1 and (F) Group 2 showing the extraction socket on PED0. Adjacent tissue sections immunostained with Periostin to identify the residual PDL in the extraction sockets of rats in (G) Group 1 and (H) Group 2. H&E-stained sagittal tissue sections on PED1 indicate granulation tissue presence in the rats of (I) Group 1 and (J) Group 2. Representative transverse histologic sections of M1 healing extraction sockets on PED7, evaluated for alkaline phosphatase (ALP) activity in (K) Group 1 and (L) Group 2 rats; dotted lines demarcate extraction site boundaries. (M) Quantification of ALP^+ve^ pixels within the region of interest for this time point. Transverse tissue sections through the extraction socket stained using Aniline blue on PED7 from rats in (N) Group 1 and (O) Group 2. (P) Quantification of Aniline Blue^+ve^ pixels/total pixels in the healing extraction site on PED7. Aniline blue-stained tissue sections on PED21 from rats in (Q) Group 1 and (R) Group 2. (S) Quantification of Aniline Blue^+ve^ pixels/total pixels in the intact alveolar bone and the healing extraction site on PED21 in both groups. Abbreviations: ab, alveolar bone; es, extraction socket; p, periodontal ligament. Scale bars = 100 μm.

To evaluate whether an osteopenic/osteoporotic phenotype affected alveolar bone healing, we created two experimental groups (Fig. 2A). In Group 1, OVX and M1 extractions were performed concurrently, and the rate of extraction socket healing was monitored. In Group 2, the OVX surgery was performed and then, after an eight-week period during which the osteoporotic phenotype developed, M1 extractions were performed (Fig. 2A). We first examined Group 2 rats to determine whether intact alveolar bone showed any overt changes in bone volume. Using a histomorphometric analysis of inter-radicular bone, no significant differences in the area of mineralized matrix/region of interest were noted (Fig. 2B, C; quantified in D).

H&E staining on tissues collected within 12 h of tooth extraction revealed a blood-filled socket in Group 1 (Fig. 2E) and a similar blood-filled socket in Group 2 (Fig. 2F). Remnants of the periodontal ligament (PDL) were visible by Periostin immunostaining in Group 1 (Fig. 2G), but the PDL remnants appeared thinner in Group 2 (Fig. 2H). Within 1 day (e.g., post-extraction day 1, PED1), the sockets in Group 1 were filled with granulation tissue (Fig. 2I), while the sockets in Group 2 were still filled with blood (Fig. 2J). On PED7, ALP staining indicated that new bone had filled the extraction sockets in samples from Group 1 (Fig. 2K; quantified in M), but extraction sockets had significantly less ALP activity in samples from Group 2 (Fig. 2L; quantified in M). New bone filled the extraction sockets in samples from Group 1 (Fig. 2N; quantified in P), but not so in samples from Group 2 (Fig. 2O; quantified in P). By PED21, the area of Aniline Blue^+ve^ bone tissue in Group 1 healed sites was equivalent to surrounding, intact bone (Fig. 2Q; quantified in S). At this time point, there was significantly less bone tissue in Group 2 extraction sites (Fig. 2R; quantified in S). Because the amount of new bone in samples from Group 2 was still significantly less than in pristine, uninjured sites (Fig. 2S), we concluded that the extraction sockets of osteoporotic rats were still in the process of healing.

### 3.3. The same drilling procedure produces a wider zone of cell death if the skeleton is osteoporotic

Cutting bone kills osteocytes [27]; we next evaluated whether an osteoporotic phenotype impacted the extent of this osteocyte cell death. Osteotomies were produced in healed extractions sites in osteoporotic rats, and in control rats. The drill diameter, drill speed, irrigation, and the site of osteotomy were identical between the two groups (see Table 1 for a complete description of the experimental cohorts).

**Fig. 3.**
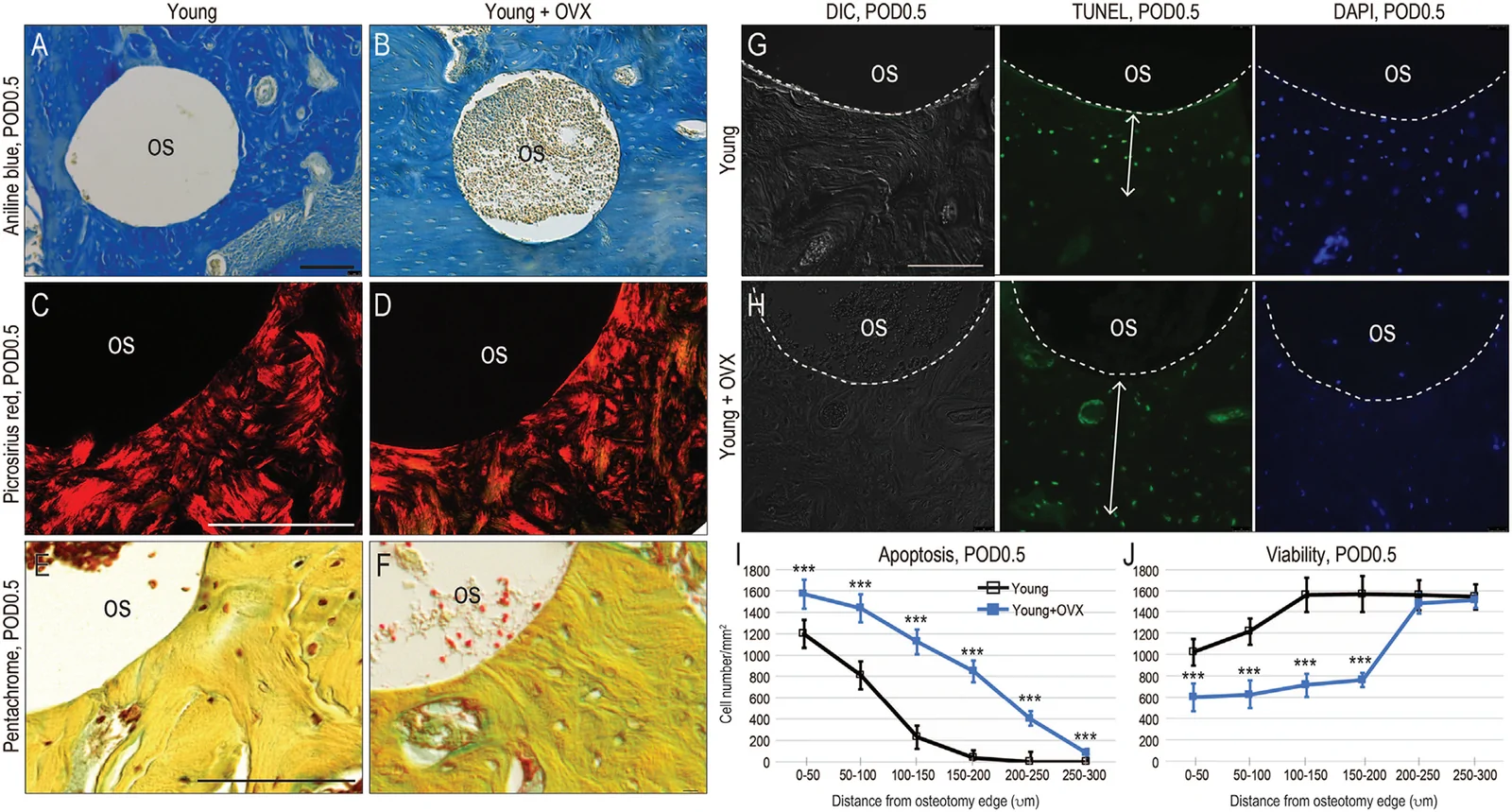
Despite an equivalent surgical procedure, significantly more osteocyte apoptosis occurs in OVX rats versus age-matched controls. Experimental model in which M1 extraction was followed by osteotomy preparation and sacrifice at post-osteotomy day 0.5 (POD0.5). Young + OVX group underwent an 8-week recovery period to allow for phenotype expression prior to osteotomy preparation and sacrifice. Aniline blue staining of transverse tissue sections from representative osteotomies prepared in (A) young and (B) young + OVX rats. Representative transverse tissue sections stained using Picrosirius Red and visualized under polarized light from (C) young and (D) young +OVX rats. High magnification of pentachrome-stained transverse tissue sections of (E) young and (F) young +OVX rats. Representative tissue sections stained using TUNEL and DAPI in (G) young and (H) young +OVX rats; dotted lines show the edge of the osteotomy. Arrows indicate the approximate extent of the radial zones of cell death. Quantification of (I) TUNEL^+ve^ apoptotic osteocyte and (J) DAPI^+ve^ viable osteocyte distribution as a function of distance from cut edge of osteotomy. Abbreviation: os, osteotomy. Scale bars = 100 μm.

Immediately after osteotomy, aniline blue staining revealed equivalently-sized, smooth-edged osteotomies in both young and young + OVX cohorts (Fig. 3A, B). Collagen organization was visualized by picrosirius red staining and when viewed under polarized light, a characteristic basket weave pattern was observed on the cut bone edges in both young and young + OVX cohorts (Fig. 3C, D). Pentachrome staining verified comparable cut surfaces in both groups (Fig. 3E, F).

The distribution of viable, dead, and dying osteocytes was mapped as a function of distance from the cut edge of the osteotomy. Differential interference contrast (DIC) microscopy was used to visualize osteocyte lacunae; on the same tissue sections, TUNEL staining identified dying (apoptotic) osteocytes, and DAPI staining identified viable osteocytes. Osteotomies created in young rats produced a ~100 μm wide zone of TUNEL^+ve^ osteocytes interspersed with some viable osteocytes (Fig. 3G). The same osteotomy produced a significantly wider (~250 μm) zone of TUNEL^+ve^, DAPI^−ve^ osteocytes in the young + OVX cohort (Fig. 3H). In a 50 μm-wide zone nearest to the osteotomy edge, the young + OVX cohort had a greater number of dying cells compared to young rats (Fig. 3I, J). From 150 μm outward, there were hardly any apoptotic osteocytes in the young cohort, whereas the young + OVX cohort still had apoptotic osteocytes ~300 μm from the cut edge (Fig. 3I). From these analyses, it was clear that the zone of apoptotic osteocytes produced by osteotomy was significantly larger in the osteopenic/osteoporotic group.

### 3.4. Finite element model predicts larger apoptotic area in osteoporotic bones

**Fig. 4.**
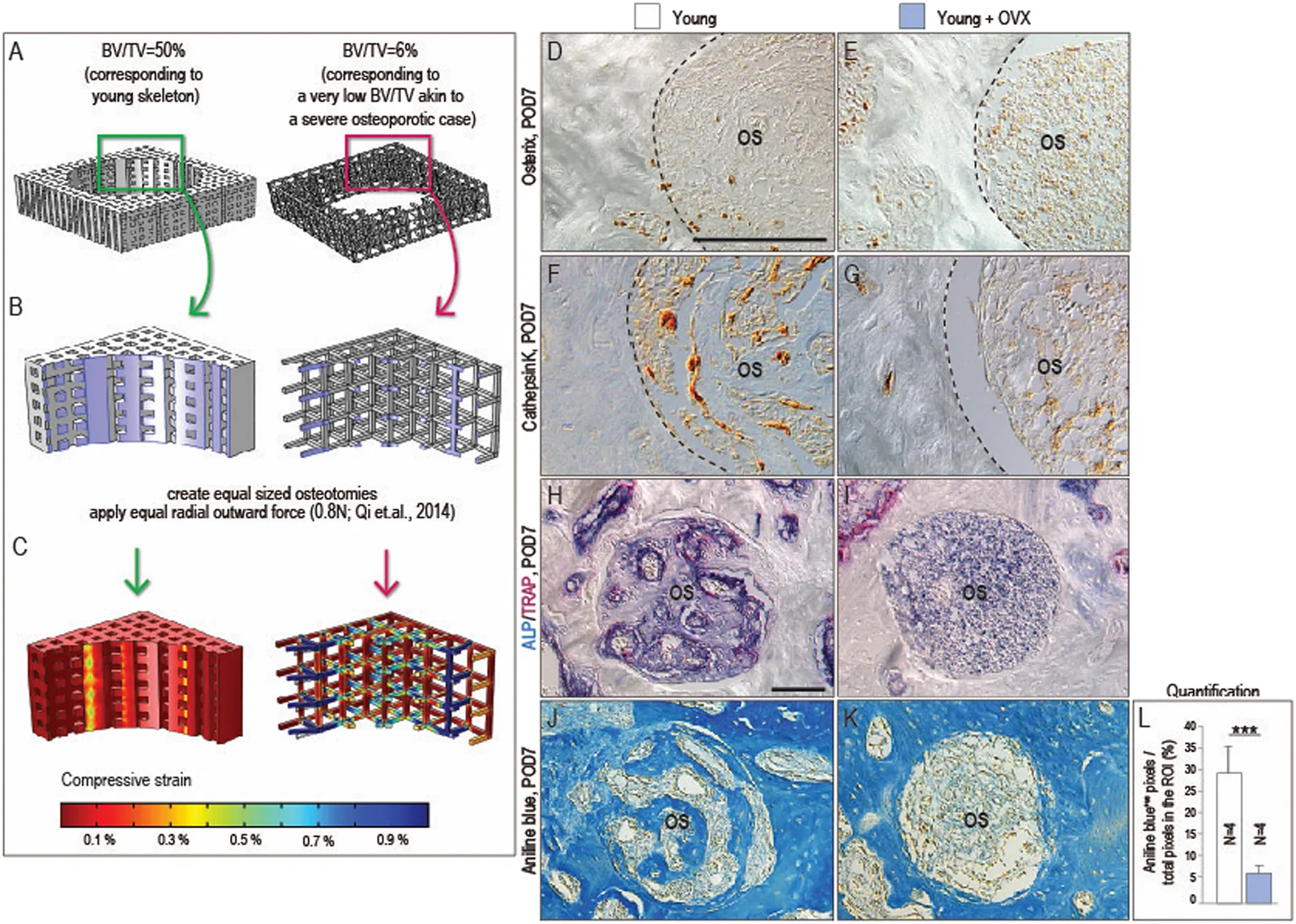
Effects of compression strain by osteotomy and alveolar bone healing of osteotomy site on an osteoporotic skeleton. (A) Representative computer-generated skeletons to model osteotomy into trabeculae of young and osteoporotic bones for finite element (FE) analysis. (B) FE model shows a radial outward force as a result of drilling into trabeculae of young and osteoporotic bones. This force is distributed along the surfaces of bony trabeculae (shown in blue) near osteotomy. (C) FE analysis shows compressive strain in trabeculae of young and osteoporotic bones during drilling. Representative transverse tissue sections from POD7, immunostained with Osterix, as a marker of osteoblast differentiation in the osteotomy sites of (D) young and (E) young +OVX rats; dotted lines show the edge of the osteotomy. Adjacent tissue sections immunostained with CathepsinK, as an indicator of osteoclast activity in the osteotomy sites (F) young and (G) young +OVX rats; dotted lines show the edge of the osteotomy. Co-staining of representative transverse tissue sections of healing osteotomy site evaluated for TRAP and ALP activity in (H) young and (I) young + OVX rats. Aniline blue-stained tissue sections evaluated for (J) young and (K) young +OVX rats. (L) Quantification of Aniline Blue^+ve^ pixels/total pixels in the osteotomy site. Abbreviation: os, osteotomy. Scale bars = 100 μm.

In an attempt to understand why site preparation in an osteoporotic animal resulted in significantly greater cell death, we evaluated the mechanical impact of drilling on young and osteoporotic bones. The major variance between the finite element (FE) model of young and young + OVX bone was the BV/TV, which was taken the literature [28]. In modeling the young cohort, a BV/TV of 50% was used whereas modeling the young + OVX cohort used a BV/TV of 6% (Fig. 4A). To simulate the effect of drilling [29] the bony trabeculae lining the cut surface of the osteotomy were subjected to an outwardly-directed radial force of 0.8 N (indicated in blue, Fig. 4B). Results from modeling the young cohort indicated minimal compressive strains on each trabeculae (i.e., 0.54%, Fig. 4C). Because the trabeculae were thinner and fewer, compressive strains on each trabeculae in the young + OVX cohort were much greater (i.e., 8%, Fig. 4C) and several times larger than the yield strain of bone. These FE results are consistent with a model whereby an osteoporotic skeleton sustains greater damage than does a healthy skeleton in response to the same mechanical force [30,31].

### 3.5. Ovariectomy negatively affects bone formation in an osteotomy

The more extensive osteocyte death observed in the osteopenic/osteoporotic cohort correlated with slower, new bone formation. For example, on POD7 there appeared to be fewer osteogenic precursors (Fig. 4D, E), and less osteoclast activity (Fig. 4F, G) in the young + OVX cohort compared to young controls. Qualitatively, the extent of ALP and TRAP staining was also less in the young + OVX cohort (Fig. 4H, I), which suggested that bone remodeling was less robust. Quantification of the amount of reparative bone in the osteotomy revealed significantly less new mineralized matrix in the osteoporotic group (Fig. 4J–L). Collectively, these data demonstrated that an osteopenic/osteoporotic phenotype showed greater damage than a young skeleton in response to the same mechanical force, which resulted in a delay in alveolar bone healing, in both extraction sites and in osteotomies produced in healed extraction sites.

## 4. Discussion

### 4.1. Aging, bone health, and healing potential

As we age, molecular and cellular damage accrues and coupled with the loss of tissue-specific progenitor cells, a functional decline in tissue homeostasis ensues [32,33]. For example, bone repair is slower in an aged population [34] but osteopenia/osteoporosis also increases with age, which makes it difficult to discriminate between the effects of aging on bone healing [4] versus the effects of low bone mass disease on bone healing. In an experimental setting, one can remove the aging variable from investigation. Here, we achieved this by producing an osteoporotic phenotype in young rats, then compared their rate of bone repair against a group of age-matched young rats. This allowed us to effectively test whether low bone mass conditions directly impacted the rate of alveolar bone healing without the confounding variable of chronologic age.

### 4.2. OVX-induced osteoporosis affects both long bones and alveolar bones

OVX results in a significant loss in trabecular number and a significant increase in trabecular separation, most notably in long bones [7]. These changes are similar to age-related alterations in the skeleton [4]. OVX only slightly altered trabecular number in alveolar bone of the same animal (Fig. 1). The evaluation of BV/TV took place ~8 weeks after surgery, as per established protocols (see [35–38]). This timeframe might not be long enough for an osteoporotic phenotype to fully develop in alveolar bones; alternatively, it could indicate that alveolar bone is somehow “resistant” to osteoporotic changes [39–41].

We evaluated alveolar bone using additional parameters and found a significant reduction in alveolar bone height (Fig. 1), similar to what has been reported in aged patients [42]. We also found that OVX rats had significantly slower extraction socket healing (Fig. 2), which would argue against a resistance hypothesis. The surgery itself was eliminated as a contributing factor to slower healing because both Groups 1 and 2 underwent OVX. The only remaining variable was that in Group 2, the osteoporotic phenotype was allowed to develop for 8 weeks before tooth extraction was performed (Fig. 2). Consequently, we can conclude that lack of estrogen due to OVX is associated with slower bone repair.

### 4.3. Potential implications of these data for patients with sub-optimal bone health

Our experimental data and computational modeling predict that, all other variables being equal, creating an osteotomy in osteoporotic patients will result in more osteocyte death than in patients with normal bone density. The greater the amount of dead or dying bone, the greater is the extent of bone resorption [21,27]. Therefore, our results here suggest that patients with uncontrolled osteoporosis may show a greater degree of bone remodeling compared to an equivalent patient population with normal bone density. If true, these data have direct clinical relevance: most dental implant patients are over age 50 [18,43], and this age group is typically osteopenic or osteoporotic [19]. While these inferences for patients remain speculative, it is nonetheless clear that methods to reduce osteocyte apoptosis caused by osteotomy site preparation will be of considerable value to any patient, especially those with sub-optimal bone health.

## Figures and Tables

**Table 1:** Experimental groups.

Group (figure #)	Procedure	Waiting period after OVX	Characteristics	Sample size

Young (Fig. 1)	None	N/A^a^	Young rats Normal bone density	*N* = 10
Young + OVX (Fig. 1)	OVX	8 weeks	Young rats Osteoporotic bone	*N* = 10
Aged (Fig. 1)	None	N/A	Aged rats Osteoporotic bone	*N* = 10
Group 1 (Fig. 2)	OVX, M1 extraction	No waiting	Young rats Normal bone density	*N* = 30
Group 2 (Fig. 2)	OVX, M1 extraction	8 weeks	Young rats Osteoporotic bone	*N* = 30
Young (Figs. 3, 4)	M1 extraction, osteotomy	N/A	Young rats Normal bone density	*N* = 12
Young + OVX (Figs. 3, 4)	OVX, M1 extraction, osteotomy	8 weeks	Young rats Osteoporotic bone	*N* = 12

a N/A = not applicable.
